# Nitrite of Amyl in Chloroform Poisoning

**Published:** 1891-10

**Authors:** E. Mammen

**Affiliations:** Bloomington, Ill.


### Nitrite of Amyl in Chloroform Poisoning.

BY E. MAMMEN, M.D., BLOOMINGTON, ILL.

I desire to add another case of chloroform poisoning treated successfully by the inhalation of nitrite of amyl. The facts are as follows: At about 7 P.M. of March 13, 1890, I received a telephone message to come hastily to M. P——, who had taken chloroform and could not be awakened. The distance was about one and a half miles, and I stopped at a drug store to procure five-drop pearls of nitrite of amyl. Time of arrival was about half an hour after being called. I found the patient in a profound stupor, respirations shallow, pulse rapid and feeble. A three ounce bottle was found in his coat pocket half full of Squibb's chloroform. A telephone message to the druggist revealed the fact that he had purchased three ounces of the drug some two hours before. He had swallowed apparently about one and a half ounce. Air was at once freely admitted to the room and to the patient, and a pearl of the nitrate given by inhalation. The effect was immediate and apparent. After the lapse of fifteen minutes, pulse again became rapid and feeble, and another pearl was used, with the result of deepening the respirations and increasing the vigor of the pulse. The same thing was repeated at lengthening intervals eight or nine times. Meanwhile hypodermic injections of atropia were twice given and towels wrung out of cold water dashed upon the chest. After four hours the patient awoke from his stupor and in another hour was out of danger. Recovery was somewhat slow, owing no doubt partly to the great destruction of red blood-corpuscles, as evidenced by the extreme icteric hue of the skin, which persisted for two weeks. In my judgment this patient could not possibly have survived without the use of nitrite of amyl.

INEBRIETY AND MEDICAL PRACTICE.—The secretary of the State Board of Health of Iowa announces that he is convinced that habitual drunkenness constitutes "palpable evidence of incompetency," as the law reads, and that therefore the physician given up to inebriety should be deprived of his certificate entitling him to practice in that State.—*Med. Record.*

